# Three-Dimensional Microwave Head Imaging with GPU-Based FDTD and the DBIM Method

**DOI:** 10.3390/s22072691

**Published:** 2022-03-31

**Authors:** Pan Lu, Panagiotis Kosmas

**Affiliations:** Faculty of Natural and Mathematical Sciences, King’s College London, Strand, London WC2R 2LS, UK

**Keywords:** distorted Born iterative method (DBIM), microwave imaging, finite-difference time-domain (FDTD), graphic processing unit (GPU), inverse scattering

## Abstract

We present a preliminary study of microwave head imaging using a three-dimensional (3-D) implementation of the distorted Born iterative method (DBIM). Our aim is to examine the benefits of using the more computationally intensive 3-D implementation in scenarios where limited prior information is available, or when the target occupies an area that is not covered by the imaging array’s transverse planes. We show that, in some cases, the 3-D implementation outperforms its two-dimensional (2-D) counterpart despite the increased number of unknowns for the linear problem at each DBIM iteration. We also discuss how the 3-D algorithm can be implemented efficiently using graphic processing units (GPUs) and validate this implementation with experimental data from a simplified brain phantom. In this work, we have implemented a non-linear microwave imaging approach using DBIM with GPU-accelerated FDTD. Moreover, the paper offers a direct comparison of 2-D and 3-D microwave tomography implementations for head imaging and stroke detection in inhomogenous anatomically complex numerical head phantoms.

## 1. Introduction

Microwave medical imaging (MWI) is gaining increased research interest [1,2,3,4,5] for its potential to offer low-cost, portable solutions to important healthcare needs such as early screening for breast cancer or detecting acute stroke inside an ambulance. Microwave tomography (MWT) is an important MWI technique which estimates the spatial distribution of dielectric properties in a reconstruction region by solving the electromagnetic (EM) inverse scattering problem [1]. EM inverse scattering processes scattered signals from the imaging domain to estimate the spatial distribution of its complex permittivity (i.e., dielectric constant and conductivity).

MWT relies on the difference in the dielectric properties of the different human tissues [6,7]. In breast imaging, for example, the dielectric contrast of cancerous vs. healthy tissue depends on the density of the tissue surrounding the tumor, and several studies have been published recently to assess this contrast with different methodologies [8,9,10,11,12,13]. High-frequency EM waves increase resolution but attenuate quickly, while low frequencies result in good penetration depth but low resolution. This trade-off dictates the working frequency range (e.g., 0.5–2.0 GHz for head imaging systems [14]).

It is well known that the inverse scattering problem of MWI is intrinsically nonlinear, and this may lead to false solutions due to the existence of local minima [15,16]. The non-linearity of the problem is combined with limited knowledge of prior information and a limited number of observation data points compared to the number of unknowns. Moreover, measurement noise and a mismatch between the model used in the imaging algorithm and the true experiment present additional challenges towards an accurate solution. Non-linearity, ill-posedness, and limited prior information in MWT require advanced optimization and regularization techniques with a careful selection of parameters that are problem-specific.

A few MWT experimental prototypes have been developed in the last twenty years [17,18,19,20,21,22], and various methods have been applied to the resulting EM inverse scattering problem [23]. Most of the work, however, has focused on two-dimensional (2-D) algorithms to avoid the added complexity of three-dimensional (3-D) implementations.

MWT 3-D implementations for medical imaging have been proposed in the literature for a few years now, but to the best of the authors’ knowledge, they have been tested primarily with simulation data [24,25,26,27,28]. A notable exception is the 3-D MWT algorithm in [2], which produced clinical 3-D microwave tomographic images of the breast by combining the discrete dipole approximation (DDA) as the forward solver with the Gauss-Newton (GN) algorithm.

MWT 2-D problems are easier to model and solve numerically, and they can often produce reconstructions of sufficient quality. In contrast, 3-D algorithms can be computationally expensive and require solving a more severely ill-posed inverse problem due to the much higher ratio of unknowns relative to data points. In iterative GN implementations such as the distorted Born iterative method (DBIM), a 3-D forward scattering problem must be solved at each iteration. This can be computationally prohibitive without the use of semi-analytical approaches [2] or numerical methods implemented in high performance hardware such as graphic processing units (GPUs) [25,29]. CPU parallelization can also model 3-D MWI forward solvers efficiently with CG type inverse solvers as in [30], where the finite element method (FEM) was combined with the contrast source inversion (CSI) method to image an experimental breast phantom, and in [31], which uses FDTD as a forward solver for reconstructing a weak scatterer. The latter is implemented on a parallel Linux cluster with a 20-core CPU and a running time of 1 h for each iteration is required.

For microwave head imaging, studies have analysed scattering from 3-D head models using FDTD [32] or FEM [33], and the FFT-based volume integral equation (VIE) method [29]. Most imaging algorithms are 2-D with only a few papers presenting 3-D reconstructions. For example, a 2-D Newton-type method using S-parameters for inversion has been proposed and tested with a experimental SAM phantom [34]. A frequency-domain beamforming imaging algorithm with Bessel function has been applied to qualitative imaging of a realistic human head in 2-D [35]. This proposed method has been further applied to 3-D head imaging using a wearable EM cap with 16 planar antennas [36] and compared with 2-D results obtained by the polar sensitivity encoding method [37] that works on the principle of encoding S-parameters. A GN-type algorithm using the open source FreeFEM++ solver has been implemented for imaging a numerical head phantom, which solves the inverse problem by considering five “sub-problems” of cross-sections in parallel across five rings of the antenna array [38]. Recently, an FEM-based and FDTD-based iterative algorithm have been implemented on a single GPU card with Tikhonov regularization and a gradient-based inverse solver [4]. This work has performed 2-D and 3-D reconstructions with both a numerical and an anthropomorphic mannequin head phantom, using a matching medium with permittivity comparable to the brain material. 3-D imaging has also been proposed in [39], which combined FEM with TSVD for linear inversion.

It is important to note that the complex structure of the head cannot be known a priori, resulting in a highly non-linear and ill-posed EM inverse scattering problem. Assuming that some prior information may be available, it is therefore important to investigate performance under different imaging scenarios. To this end, we have recently applied the DBIM with the fast iterative shrinkage/thresholding algorithm (FISTA) [40] to 2-D numerical brain phantoms with limited prior information. Results showed that it is possible to reconstruct the target inside a head phantom of known shape with several unknown tissue layers [41].

This work investigates microwave head imaging further with the help a 3-D DBIM algorithm. This allows to test performance for more realistic scenarios, where weak target responses are captured by a limited number of antennas surrounding the head in a 3-D array. To this end, we have implemented an in-house GPU-based 3-D FDTD forward solver, which enables a computationally efficient 3-D implementation of our recently proposed DBIM-FISTA. By optimizing the FDTD code specifically for our application, we achieve a more computationally efficient implementation than previous work [3,25] which used GPU-based platform Acceleware [42] to perform MWT reconstructions of numerical breast phantoms of varying tissue density.

We also simplify the 3-D inversion further by considering a scalar Green’s function, which assumes a single electric field component transmission by our linearly polarized antenna. We compare results from this approximation with the vectorial Green’s function which considers all three electric field components inside the reconstruction domain. Importantly, our results show, for the first time in microwave head imaging, that the scalar 3-D approximation leads to almost identical results with the vectorial implementation. Our DBIM algorithm is also more efficient at each DBIM iteration, as the employed FISTA algorithm uses an accelerated momentum which leads to faster convergence than CG-type inverse solvers [43]. Moreover, as a shirnkage/thresholding algorithm, it helps to reduce the chance of getting stuck into local minima [44].

The algorithm is tested on different reconstruction scenarios with the MRI-based Zubal head phantom [45] and is validated with an experimental brain phantom. Beyond validating the algorithm, our study examines the benefits of imaging in 3-D by comparing with 2-D implementations for challenging target location and limited prior information of the head’s structure. To this end, we use a cross-sectional axial slice from the 3-D brain model and compare reconstructed images for this slice using the 2-D and 3-D algorithm.

The remainder of the paper is organized as follows: In Section 2, we review our recently proposed DBIM-FISTA algorithm [40] for reconstructing the spatial distribution of dielectric properties inside the region of interest. We also presents the algorithm’s implementation in 3-D with our in-house, GPU-based FDTD forward solver. It also details the experimental head phantom and the different head models based on the Zubal head phantom used to test our algorithm. Reconstruction results for the numerical models and experimental phantom are presented in Section 3, followed by discussion in Section 4.

## 2. Materials and Methods

### 2.1. The DBIM-FISTA Algorithm

The DBIM can solve nonlinear EM inverse scattering problems iteratively to reconstruct the spatial distribution of dielectric properties within a region *V* [25]. It is based on approximating the non-linear integral equation which describes the relationship of the electric field with the continuous spatial distribution of dielectric properties via the Born approximation. The non-linear integral equation of the fields scattered by the object to be imaged for each transmitter-receiver (TR) pair can be written as,

(1) $${\bar{E}}_{s}\left(r_{n},r_{m}\right)={\bar{E}}_{t}\left(r_{n},r_{m}\right)-{\bar{E}}_{b}\left(r_{n},r_{m}\right)=\omega^{2}\mu_{0}\varepsilon_{0}\int_{V}{\bar{G}}_{b}\left(r_{n},r\right){\bar{E}}_{t}\left(r,r_{m}\right)\delta_{\varepsilon }\left(r\right)dr,$$

where ${\bar{E}}_{t}$, ${\bar{E}}_{s}$, ${\bar{E}}_{b}$ are the total, scattered and background vector electric fields, respectively. The total field is measured at each antenna, but is unknown inside region *V*, and is approximated by $E_{b}$ under the Born approximation. The vectors $r_{m}$ and $r_{n}$ denote the transmitting and receiving antenna locations, ω is the angular frequency, $\mu_{0}$ and $\varepsilon_{0}$ are the permeability and permittivity of free space, and ${\bar{G}}_{b}$ is the dyadic Green’s function for the background medium. The difference $\delta_{\varepsilon }$ between the relative complex permittivities of reconstruction ε(r) and background $\varepsilon_{b}\left(r\right)$ is defined as

(2) $$\delta_{\varepsilon }\left(r\right)=\varepsilon_{r}\left(r\right)-\varepsilon_{b}\left(r\right).$$

Assuming an equal discretization step Δh for each dimension and constant permittivity inside each voxel, (1) can be written as a discrete equation as,

(3) $${\bar{E}}_{s}\left(r_{n},r_{m}\right)=k_{0}^{2}\Delta h^{3}\sum_{r\in V}{\bar{G}}_{b}\left(r_{n},r\right){\bar{E}}_{b}\left(r,r_{m}\right)\delta_{\varepsilon }\left(r\right),$$

where

(4) $${\bar{E}}_{s}\left(r_{n},r_{m}\right)=\left[\begin{array}{c} E_{s}^{x}\left(r_{n},r_{m}\right)\\ E_{s}^{y}\left(r_{n},r_{m}\right)\\ E_{s}^{z}\left(r_{n},r_{m}\right) \end{array}\right],\;{\bar{E}}_{b}\left(r,r_{m}\right)=\left[\begin{array}{c} E_{x}\left(r,r_{m}\right)\\ E_{y}\left(r,r_{m}\right)\\ E_{z}\left(r,r_{m}\right) \end{array}\right],$$

with $E_{x}$, $E_{y}$, $E_{z}$ representing the *x*-, *y*-, *z*-direction fields of the background field ${\bar{E}}_{b}$ respectively, and $k_{0}$ is the free-space wavenumber.

The relation between the background field ${\bar{E}}_{b}$ and the Green’s function ${\bar{G}}_{b}$ due to a source with current density ${\bar{J}}_{0}$ at $r_{0}$ holds as [46],

(5) $$\begin{array}{cc} {\bar{E}}_{b}\left(r,r_{0}\right) & =-j\omega \mu_{0}\int_{V}{\bar{J}}_{0}\left(r^{\prime }\right)\cdot {\bar{G}}_{b}\left(r^{\prime },r\right)dr^{\prime }\\ \left[\begin{array}{c} E_{x}\left(r,r_{0}\right)\\ E_{y}\left(r,r_{0}\right)\\ E_{z}\left(r,r_{0}\right) \end{array}\right] & =-j\omega \mu_{0}\int_{V}\left[\begin{array}{c} J_{x}\left(r^{\prime }\right)\\ J_{y}\left(r^{\prime }\right)\\ J_{z}\left(r^{\prime }\right) \end{array}\right]\cdot \left[\begin{array}{c} G_{xx}\left(r^{\prime },r\right),G_{xy}\left(r^{\prime },r\right),G_{xz}\left(r^{\prime },r\right)\\ G_{yx}\left(r^{\prime },r\right),G_{yy}\left(r^{\prime },r\right),G_{yz}\left(r^{\prime },r\right)\\ G_{zx}\left(r^{\prime },r\right),G_{zy}\left(r^{\prime },r\right),G_{zz}\left(r^{\prime },r\right) \end{array}\right]dr^{\prime }. \end{array}$$

where $J_{x}$, $J_{y}$ and $J_{z}$ are the *x*-, *y*- and *z*-components of the source current density. When an antenna due to a single polarization point source current $I_{z}$ (or $I_{x}$ or $I_{y}$) inside a voxel with length Δh is used,

(6) $$J_{i}\left(r\right)=I_{i}\Delta h\delta \left(r\right)\;\mathrm{and}\;{\bar{G}}_{b}=\left\{G_{ij}\right\},$$

where i,j=x,y or *z*, $I_{i}$ is the transmitting current at the antenna, and δ(r) is the Dirac delta function. In this case, the background dyadic Green’s function is linked to the background electric field as [25,47],

(7) $${\bar{G}}_{b}\left(r_{0},r\right)=\frac{j}{\omega_{0}\mu_{0}\Delta h}\left[\begin{array}{ccc} \frac{1}{I_{x}}E_{x}\left(r,r_{0}\right) & \frac{1}{I_{x}}E_{y}\left(r,r_{0}\right) & \frac{1}{I_{x}}E_{z}\left(r,r_{0}\right)\\ \frac{1}{I_{y}}E_{x}\left(r,r_{0}\right) & \frac{1}{I_{y}}E_{y}\left(r,r_{0}\right) & \frac{1}{I_{y}}E_{z}\left(r,r_{0}\right)\\ \frac{1}{I_{z}}E_{x}\left(r,r_{0}\right) & \frac{1}{I_{z}}E_{y}\left(r,r_{0}\right) & \frac{1}{I_{z}}E_{z}\left(r,r_{0}\right) \end{array}\right].$$

Note that the current terms in (7) correspond to excitation currents for the transmitting antennas. Hence, only the last row of the matrix is non-zero for a *z*-polarized antenna, leading to a Green’s function that is reduced to [25,47],

(8) $${\bar{G}}_{b}\left(r_{0},r\right)=\frac{j}{\omega_{0}\mu_{0}\Delta h}\left[\begin{array}{ccc} 0 & 0 & 0\\ 0 & 0 & 0\\ \frac{1}{I_{z}}E_{x}\left(r,r_{0}\right) & \frac{1}{I_{z}}E_{y}\left(r,r_{0}\right) & \frac{1}{I_{z}}E_{z}\left(r,r_{0}\right) \end{array}\right].$$

Using (8), the vector product of ${\bar{G}}_{b}$ and ${\bar{E}}_{b}$ in (3) is computed as,

(9) $${\bar{G}}_{b}\left(r_{n},r\right){\bar{E}}_{b}\left(r,r_{m}\right)=\frac{j}{\omega_{0}\mu_{0}I_{z}\Delta h}\left[\begin{array}{c} 0\\ 0\\ \sum_{i=x,y,z}E_{i}\left(r,r_{n}\right)E_{i}\left(r,r_{m}\right) \end{array}\right].$$

As our antenna records the *z*-component of the electric field, a scalar approximation of the scattered field

(10) $${\bar{E}}_{s}={\bar{E}}_{s}\cdot \hat{z}=E_{z}^{s}$$

in the LHS of (3) can be used [25,47,48]. Thus (3) is further reduced to a scalar equation as,

(11) $$E_{z}^{s}=k_{0}^{2}\Delta h^{3}\sum_{r\in V}\left[{\bar{G}}_{b}\left(r_{n},r\right){\bar{E}}_{b}\left(r,r_{m}\right)\delta_{\varepsilon }\left(r\right)\right]\cdot \hat{z},$$

where the *z*-component of product (9) is computed as,

(12) $${\bar{G}}_{b}^{z}\left(r_{n},r\right){\bar{E}}_{b}\left(r,r_{m}\right)=\frac{j}{\omega_{0}\mu_{0}I_{z}\Delta h}\sum_{i=x,y,z}E_{i}\left(r,r_{n}\right)E_{i}\left(r,r_{m}\right)$$

and

(13) $${\bar{G}}_{b}^{z}\left(r_{n},r\right)=\frac{j}{\omega_{0}\mu_{0}\Delta hI_{z}}\left[\begin{array}{ccc} E_{x}\left(r,r_{n}\right) & E_{y}\left(r,r_{n}\right) & E_{z}\left(r,r_{n}\right) \end{array}\right].$$

If we assume that the cross-products of *x*- and *y*-components are negligible [25] when a *z*-polarized antenna is used, we can simplify the Green’s function further to account only for the *z*-directed components as

(14) $$G_{b}\left(r_{n},r\right)=\frac{j}{\omega_{0}\mu_{0}I_{z}\Delta h}E_{z}\left(r,r_{n}\right).$$

We must emphasize that the simplification of assuming a *z*-polarized source is only done by our imaging algorithm, and is not used to generate the data in CST, which models the exact antenna used in our experiments. Moreover, the assumption that only the *z*-component of the Green’s function is non-zero is employed only in the inversion of the linear matrix at each DBIM iteration. The 3D FDTD forward solver used by the imaging algorithm can calculate all three field components. This allows comparing the *z*-only approximation of the Green’s function with the vector formulation in (13). We have compared results from these two formulations for all our imaging scenarios and concluded that the scalar approximation produced results of similar quality. More details on this important contribution of this work are presented in Section 3.

By substituting the Green’s function in (3), the ill-posed linear system is built as,

(15) $$b=A\delta_{\varepsilon },$$

where A={A(p,q)} is an M×N matrix (M≪N), with *M* transmit-receive pairs and *N* voxels of the reconstruction region *V*,

(16) $$A\left(p,q\right)=k_{0}^{2}\Delta h^{3}{\bar{G}}_{b}\left(r_{n},q\right){\bar{E}}_{b}\left(q,r_{m}\right),$$

*p* is the *p*-th TR pair ${\left(r_{n},r_{m}\right)}_{p}$, *q* is the *q*-th voxel inside region *V*, and $b=\{E_{s}\left(p\right)\}$ is a M×1 vector of the scattered fields. The computational complexity of (16) using the dyadic Green’s function is O(M×9N), while the computational complexity further reduced to O(M×3N) and O(M×N) respectively when using (13) and (14). At each iteration *K*, the forward solver (FDTD for our implementation) is run first to obtain the background data and then used to build (15), which is solved by the inverse solver FISTA. Finally, the background profile is updated by

(17) $$\varepsilon_{b}^{K+1}\left(r\right)=\varepsilon_{b}^{K}\left(r\right)+\delta_{\varepsilon }\left(r\right)$$

and the DBIM continues to next iteration K+1.

We use a single-pole Debye model to simulate the frequency-dependent materials in FDTD as,

(18) $$\varepsilon_{r}\left(\omega \right)=\varepsilon_{\infty }+\frac{\Delta \varepsilon }{1+j\omega \tau }+\frac{\sigma_{s}}{j\omega \varepsilon_{0}},$$

where $\varepsilon_{\infty }$, Δε, τ and $\sigma_{s}$ are the parameters of the Debye model. Thus (15) is constructed as,

(19) $$\left({A^{ℜ}+jA}^{ℑ}\right)\left(\delta_{\varepsilon_{\infty }}+\frac{\delta_{\Delta \varepsilon }}{1+j\omega \tau }+\frac{\delta_{\sigma_{s}}}{j\omega \varepsilon_{0}}\right)=b^{ℜ}+jb^{ℑ},$$

where *ℜ* and *ℑ* represents the real and imaginary part respectively and

(20) $$A=A^{ℜ}+jA^{ℑ}.$$

As we only consider real valued computations, the following linear system is built as,

(21) $$\left[\begin{array}{c} b^{ℜ}\left(1\right)\\ b^{ℑ}\left(1\right)\\ b^{ℜ}\left(2\right)\\ b^{ℑ}\left(2\right)\\ \ldots \\ b^{ℜ}\left(M\right)\\ b^{ℑ}\left(M\right) \end{array}\right]=\left[\begin{array}{c} {\bar{A}}_{\varepsilon_{\infty }}^{ℜ}\left(1\right),{\bar{A}}_{\Delta \varepsilon }^{ℜ}\left(1\right),{\bar{A}}_{\sigma_{s}}^{ℜ}\left(1\right)\\ {\bar{A}}_{\varepsilon_{\infty }}^{ℑ}\left(1\right),{\bar{A}}_{\Delta \varepsilon }^{ℑ}\left(1\right),{\bar{A}}_{\sigma_{s}}^{ℑ}\left(1\right)\\ {\bar{A}}_{\varepsilon_{\infty }}^{ℜ}\left(2\right),{\bar{A}}_{\Delta \varepsilon }^{ℜ}\left(2\right),{\bar{A}}_{\sigma_{s}}^{ℜ}\left(2\right)\\ {\bar{A}}_{\varepsilon_{\infty }}^{ℑ}\left(2\right),{\bar{A}}_{\Delta \varepsilon }^{ℑ}\left(2\right),{\bar{A}}_{\sigma_{s}}^{ℑ}\left(2\right)\\ \ldots ,\ldots ,\ldots \\ {\bar{A}}_{\varepsilon_{\infty }}^{ℜ}\left(M\right),{\bar{A}}_{\Delta \varepsilon }^{ℜ}\left(M\right),{\bar{A}}_{\sigma_{s}}^{ℜ}\left(M\right)\\ {\bar{A}}_{\varepsilon_{\infty }}^{ℑ}\left(M\right),{\bar{A}}_{\Delta \varepsilon }^{ℑ}\left(M\right),{\bar{A}}_{\sigma_{s}}^{ℑ}\left(M\right) \end{array}\right]\left[\begin{array}{c} \delta_{\varepsilon_{\infty }}\left(1\right)\\ \delta_{\varepsilon_{\infty }}\left(2\right)\\ \ldots \\ \delta_{\varepsilon_{\infty }}\left(N\right)\\ \delta_{\Delta \varepsilon }\left(1\right)\\ \delta_{\Delta \varepsilon }\left(2\right)\\ \ldots \\ \delta_{\Delta \varepsilon }\left(N\right)\\ \delta_{\sigma_{s}}\left(1\right)\\ \delta_{\sigma_{s}}\left(2\right)\\ ...\\ \delta_{\sigma_{s}}\left(N\right) \end{array}\right],$$

where

(22) $${\bar{A}}_{\varepsilon_{\infty }}^{ℜ}=A^{ℜ},{\bar{A}}_{\varepsilon_{\infty }}^{ℑ}=A^{ℑ},{\bar{A}}_{\Delta \varepsilon }^{ℜ}=\frac{A^{ℜ}+\omega \tau A^{ℑ}}{1+\omega^{2}\tau^{2}},{\bar{A}}_{\Delta \varepsilon }^{ℑ}=\frac{A^{ℑ}-\omega \tau A^{ℜ}}{1+\omega^{2}\tau^{2}},{\bar{A}}_{\sigma_{s}}^{ℜ}=\frac{A^{ℑ}}{\omega \varepsilon_{0}},{\bar{A}}_{\sigma_{s}}^{ℑ}=\frac{-A^{ℜ}}{\omega \varepsilon_{0}}.$$

We also use the convolutional perfectly matched layer (CPML) absorbing boundary condition to terminate the FDTD computational domain.

A minimization problem with a regularization term is considered by FISTA as the following steps when $l_{1}$ norm is chosen,

(23) $$F(x)=\frac{1}{2}{∥\mathrm{Ax}-b∥}_{2}^{2}+\lambda {∥x∥}_{1}$$

where λ is a regularization parameter. Thus the structure of FISTA is constructed as [43],

(24) $$\begin{array}{c} x_{k}=p_{L_{k}}\left(y_{k}\right)=\psi \left(y_{k}-\frac{1}{L_{k}}A^{T}\left(Ay_{k}-b\right)\right),\\ y_{k+1}=x_{k}+\frac{t_{k}-1}{t_{k+1}}\left(x_{k}-x_{k-1}\right) \end{array}$$

where

(25) $$t_{0}=1\;\mathrm{and}\;t_{k+1}=\frac{1+\sqrt{1+4t_{k}^{2}}}{2},$$

ψ is the soft thresholding function

(26) ψ(x)=sign(x)|x−λ|,

$y_{k}$ is the solution at the *k*–th iteration. $L_{k}$ is a non-negative parameter which is selected based on the following strategy: Find the smallest non-negative integers $i_{k}$ with

(27) $$L_{k}=\eta^{i_{k}}L_{k-1}\;\mathrm{and}\;L_{0}=1,$$

such that

(28) $$F\left(p_{L_{k}}\left(y_{k}\right)\right)\leq Q_{L_{k}}(\left(p_{L_{k}}\left(y_{k}\right),y_{k}\right),$$

where

(29) $$\eta >1\;\mathrm{and}\;Q_{L}\left(x,y\right)=\frac{1}{2}{∥\mathrm{Ay}-b∥}_{2}^{2}+\langle x-y,A^{T}Ay-b\rangle +\frac{1}{2}{∥x-y∥}_{2}^{2}+\lambda {∥x∥}_{1}.$$

The computational complexity for FISTA is O(kMN) with a higher order convergence rate $O(\frac{1}{k^{2}})$ compared with the traditional CG-type algorithm. The improvement is caused by the use of the accelerated momentum $t_{k}$, which also reduces the chance of getting stuck into a local minimum when combined with a regularization term [44].

Our implementation selects the initial value of x to be 0 and the regularization parameter as

(30) $$\lambda =\delta ∥A^{T}{b∥}_{\infty },$$

where δ is a factor with

(31) 0<δ<1.

The stopping criterion is based on the relative error between current $F(x_{k})$ and previous $F(x_{k-1})$, defined as

(32) $$e_{opt}=\frac{|F\left(x_{k}\right)-F\left(x_{k-1}\right)|}{F(x_{k-1})}.$$

FISTA stops when the relative error $e_{opt}$ becomes smaller than a preset value, usually chosen between $10^{-4}$ and $10^{-2}$.

### 2.2. Implementation of the DBIM-FISTA with a GPU-Based FDTD Forward Solver

We have used CUDA toolkit to accelerate our FDTD forward solver in GPU similar to [49], which focused however on modeling RF wave interactions in high-field MRI. The FDTD algorithm consists of electric and magnetic field updates, and each can be viewed as a kernel on GPU. The kernel, executed as a grid in GPU, can be divided into multiple blocks of threads, whose number can be adjusted based on the GPU platform. We can therefore increase the computational efficiency significantly by computing the blocks of threads simultaneously. Our algorithm is designed to use the high performance of GPU without changing the MATLAB based DBIM code, to take advantage of MATLAB’s capability for matrix computing. To this end, we have implemented the 3-D FDTD algorithm in C++ with CUDA and then incorporated it with MEX functions in MATLAB, which is also used to implement the FISTA inverse solver. The environment of our implementation is MATLAB 2020 (Mathworks Inc., Natick, MA, USA), CUDA 10.2 (Nvidia Co., Santa Clara, CA, USA) and Visual Studio 2015 (Microsoft Co., Redmond, WA, USA) with Intel^®^ Xeon^®^ CPU E5-2640 v3 @ 2.60 GHz and GPU Tesla K20c with 5 GB memory.

The maximum number of threads for each block is 1024 and we use a 2-D block with size 32×32=1024 to make full use of each block. The number of threads for each block should be set to a multiple of 32 as the working unit in the GPU is a warp, which is a set of 32 threads [50]. Similarly, we use a 2-D grid GPU with size $\frac{Imax}{32}\times \frac{Jmax}{32}$ for each kernel, $E_{i}$ (i=x,y or *z*) for example, and the loop inside each kernel for updating the fields is in the following structure shown in Algorithm 1, where Imax, Jmax and Kmax represent number of voxels for the *x*, *y*, and *z* dimension, respectively.
**Algorithm 1** Kernel of updating electric fieldti←blockIdx.x*blockDim.x+threadIdx.xtj←blockIdx.y*blockDim.y+threadIdx.x**if** ti<Imax and tj<Jmax **then**   **for** tk←1 to Kmax **do**     $E_{i}\left[ti+tj*Imax+tk*\left(Imax*Jmax\right)\right]\leftarrow UpdateE_{i}$   **end for** **end if**

**Figure 1:**
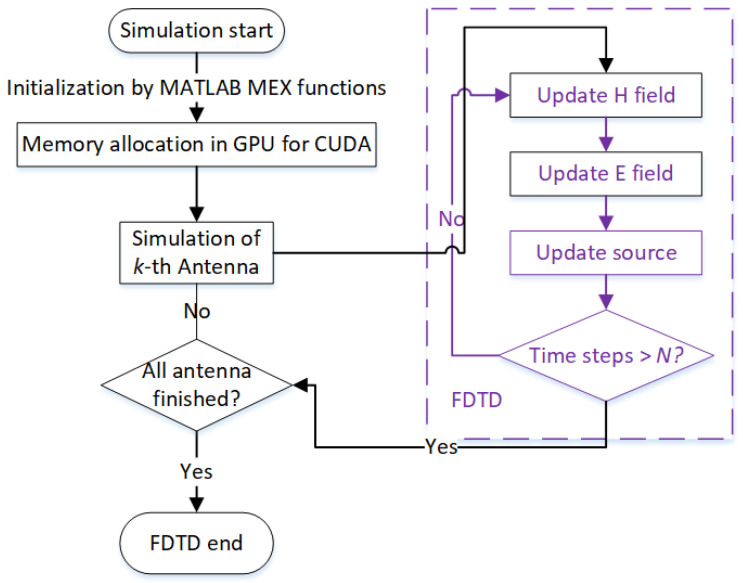
Flowchart of the GPU-based FDTD algoritm.

The GPU Tesla K20c has 13 streaming processors (SMs) and for each SM there are at most 2048 threads. Thus 13 × 2048 threads can be run simultaneously. The flowchart of the 3-D FDTD algorithm is shown in Figure 1. To reduce computational times, most of the FDTD functions are run on GPU (kernels), including the source update.

At each DBIM iteration, the FDTD variables are first allocated space on GPU after initialization by MEX functions. Thus all FDTD-related variables are on GPU memory. Then the FDTD part on GPU starts to simulate the wave propagation for each transmitter antenna, and the calculated data to be used in DBIM is transferred back to MATLAB via MEX functions, which can be used directly by the MATLAB code. Finally the ill-conditioned linear system is built by (3) and solved by FISTA to obtain the reconstructed values.

The discretization size for FDTD is defined as

(33) $$\Delta h=\frac{\lambda_{f}}{n_{d}},$$

where $\lambda_{f}$ is the wavelength and $n_{d}$ is the discretization step. To ensure accuracy and reduce numerical dispersion, $n_{d}>10$ is required. While Δh is 30 mm in air, its value inside the brain is defined by a much smaller wavelength ($\lambda_{f}=\frac{300}{\sqrt{\varepsilon_{r}}}$), thus requiring a greater $n_{d}$ and a smaller Δh. We have therefore selected a cubic grid voxel size of 2 mm for each dimension in all our 2-D and 3-D FDTD simulations. This value can balance accuracy and computational burden. For example, using a 2 mm vs. a 1 mm cubic voxel side results in field values with a mean relative difference of $10^{-3}$ but requires an 8 times bigger grid. The time step Δt defined as

(34) $$\Delta t=\frac{\Delta h}{2c},$$

where *c* is the speed of EM wave, will also increase for a higher resolution model and thus lead to more iterations. In our simulations, the iteration number ranges between 1000–2000.

To compare computational burden vs. grid resolution, we calculated run times for a two antenna system using 2 mm, 1.5 mm and 1 mm size cubic voxels. These are acceptable resolutions for a grid with physical volume of about 300 mm × 300 mm × 200 mm, which is required for our inverse problem with the numerical head phantom. Resolutions of 0.5 mm or lower exceed the GPU’s memory requirements, and they also increase errors and instabilities for the inverse model [51]. The size and run times corresponding to these different grids when 16 antennas are used as transmitters and receivers are shown in Table 1.

As this implementation is specific for the inverse problem at hand, it is more than 30% faster than our previously used codes implemented with commercial software package Acceleware, which is designed for general GPU-based FDTD simulations. Moreover, the mean absolute error of the fields received by each antenna calculated by Acceleware and our new code is less than $10^{-8}$, which shows our implementation is as accurate as Acceleware’s. The running time for FISTA for the above-mentioned case with 2 mm resolution where the size of *A* is 240 × 449,949 at each DBIM iteration, is around 5–15 s depending on the number of FISTA iterations. This number typically ranges from 20–100, i.e., for each iteration the average running time is around 0.2 s.

As noted earlier, we have also employed the 2-D DBIM-FISTA algorithm [40] in this paper to compare performance with the 3-D implementation. The transverse magnetic (TM) mode of the EM wave is considered for the 2-D problem. The 2-D code is implemented in MATLAB without the need of GPU acceleration for the FDTD forward model, which requires approximately 1 s for each of the eight transmitting antennas and a total of 10–20 s for each DBIM iteration.

### 2.3. Numerical and Experimental Phantoms

The original MRI-derived Zubal head phantom [45] comprises 256 × 256 × 128 voxels with size of 1.1 mm × 1.1 mm × 1.4 mm. First, we imported the phantom into CST Microwave Studio to obtain the numerical “measured” data (S-parameters) of the full-wave 3-D interaction of the experimental antenna array with the phantom, which is used to test the 3-D algorithm. We then resized the model to fit the FDTD’s grid of 2 mm for each side. We have also transformed the original Zubal head model from dozens of materials into an eight-material head model. The permittivity of the materials used in CST was obtained from the IT’IS foundation database [52]. These data were also used to develop single-pole Debye models for our FDTD code by curve fitting. As these two approaches are not identical, there are discrepancies between the CST and FDTD Debye models.

**Figure 2:**
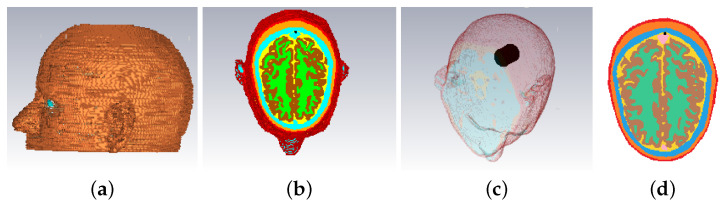
Views of the Zubal phantom in CST: (**a**) 3-D side-view, (**b**) cross-sectional top-down view, (**c**) 3-D view with blood target inserted, (**d**) cross-sectional slice used in the 2-D experiments (93th slice of the original model, where the center of the target is placed). These phantoms are used for the “Case I” imaging scenario described in Section 3.

The 3-D structure of the Zubal head phantoms in CST is shown in Figure 2.

As for the 2-D cases, a slice of the Zubal head phantom inside the brain is used in FDTD as shown in Figure 2d. The model includes eight layers with tissue types, color codes and respective Debye parameters (for our FDTD models) shown in Table 2.

The head models are surrounded by 90–10% glycerol-water mixture, which we have used previously as immersion liquid in our recent experiment work [53,54]. The permittivity of the glycerol-water mixture is $\varepsilon_{r}\approx 15.9-14.2j$ at 1.0 GHz. The Debye parameters $\varepsilon_{\infty }=6.566$, Δε=16.86 and $\sigma_{s}=0.3231$. The relaxation time τ is fixed as 0.14288 ns for all the materials.

**Figure 3:**
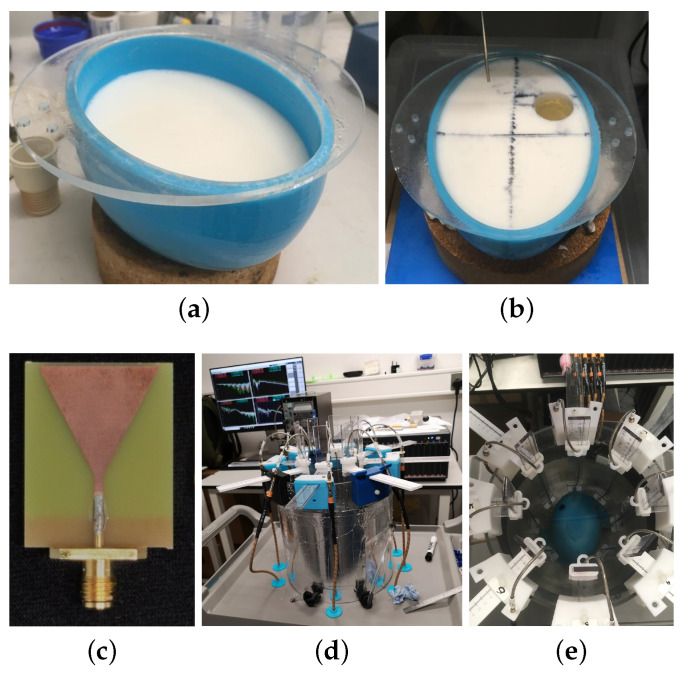
Photos of the experimental head phantom (**a**,**b**), antenna (**c**) and system (**d**,**e**) used to validate the 3-D DBIM-FISTA algorithm. Details of the setup and experiment can be found in [54].

Starting from this head model, we have studied various different imaging scenarios, which will be discussed in next section. Moreover, we have used a simplified experimental head phantom [54] shown in Figure 3 to validate our algorithm with more realistic data.

The phantom is made of a 3D-printed plastic mould and is filled with an “average brain tissue” material with $\varepsilon_{r}\approx 41.6-5.9j$ at 1.0 GHz. We should note that the permittivity of the brain tissue differs at different positions, i.e., the permittivity near the different surface, in the inner parts or near the bottom has different values [54]. The brain phantom is immersed in the 90–10% glycerol-water mixture inside an imaging tank, which is made of acrylic and is surrounded by absorbing material ECCOSORB MCS covered by a metallic shield. A printed monopole triangular patch antenna is used, as shown in Figure 3c, which operates well in the frequency range 0.5–2 GHz [55]. An eight-antenna array inside the tank captures MWT data with the help of an eight-port VNA system embedded in the Keysight M9019A PXIe Chassis connected to a desktop, shown in Figure 3d,e. Further details of the experimental system and the process of making the phantom can be found in [54].

The number of required antennas for a 2-D microwave imaging problem has been defined in [56] using the approximation,

(35) $$M\approx 2k_{0}\alpha ,$$

where α is the radius of the investigated region, while for 3-D problems the required number is slightly larger. This requirement may be difficult to satisfy in practice due to experimental limitations; for example, our experimental setup uses eight antenna hosted by an eight-port VNA system, and the same setup is used for each antenna ring in our CST scenarios. We note that the antennas are modelled as point sources in our algorithms, and the received signals by the antennas are normalized with respect to the source. The calibrated scattered fields are a standard total field calibration procedure [57] as,

(36) $$E_{cali}^{s}\left(i,j\right)=\frac{E_{b}\left(i,j\right)}{S_{b}\left(i,j\right)}\left(S_{t}\left(i,j\right)-S_{b}\left(i,j\right)\right)$$

where $S_{b}$ and $S_{t}$ represent the S-parameters for the background case and for the unknown case to be imaged, as measured experimentally or modeled in CST. In (36), $E_{b}$ represents the background incident signals calculated by the FDTD forward model, and (i,j) refers to the transmitter-receiver pair.

## 3. Results

### 3.1. Validation of the Proposed DBIM-FISTA Algorithm

This subsection presents results from simple imaging scenarios where only a stroke-like target is unknown, with the aim to validate our 3-D DBIM-FISTA implementation. We consider both CST-calculated and experimental data and compare our results with 2-D scenarios where the target is placed at the same height as the antenna ring used by the 2-D algorithm. The 2-D DBIM-FISTA can perform well for these scenarios, as it deals with a detectable scattered signal and an inverse problem of much fewer unknowns. Although the number of iterations required for convergence will be different for each 2-D and 3-D imaging scenario, we have selected a fixed number of 20 iterations for this comparison. To study the impact of keeping the number of iterations fixed, we have compared results with a much greater number of iterations for “Case I” presented in Section 3.1.1. We note that all of our reconstructions estimate 3D volume distributions, but we only show the 2D axial slice results, as we have limited array coverage along the *z*-axis, and we also want to compare directly 2D and 3D simulations along the axial planes.

**Figure 4:**
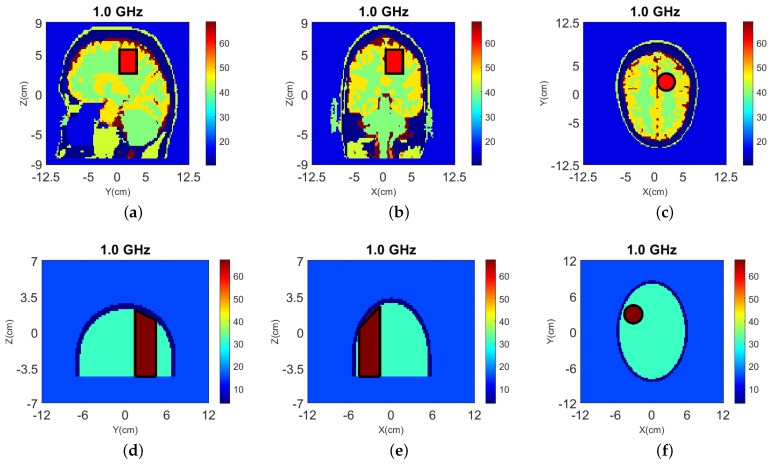
Dielectric constant distribution $ℜ(\varepsilon_{r})$ for our validation models. **Top**: (**a**) y-z slice, (**b**) x-z slice, and (**c**) x-y slice for the numerical head model; **Bottom**: (**d**) y-z slice, (**e**) x-z slice, and (**f**) x-y slice for the experimental phantom.

The true dielectric constant values $ℜ(\varepsilon_{r})$ of the numerical and experimental phantoms are shown in Figure 4.

The root mean square error (*RMSE*) of the xy-slice reconstruction contrast is used to compare the 2-D and 3-D reconstructions, defined as

(37) $$RMSE=\sqrt{\frac{1}{N_{r}}\sum_{p=1}^{N_{r}}e_{\varepsilon }^{2}\left(p\right)},$$

where $N_{r}$ is the number of voxels inside the reconstruction region, *p* is the index of the voxel, and $e_{\varepsilon }$ is the difference between the real part of relative permittivity of reconstruction $\varepsilon_{r}\left(p\right)$ and true values of $\varepsilon_{gt}\left(p\right)$, i.e.,

(38) $$e_{\varepsilon }=\varepsilon_{r}\left(p\right)-\varepsilon_{gt}\left(p\right).$$

The max error of the xy-slice reconstruction contrast is defined as

(39) $$e_{max}=max\left|e_{\varepsilon }\left(p\right)\right|,$$

and the relative error is defined as the relative norm between the residual errors at the *K*–th and first DBIM iteration,

(40) $$RE=\frac{∥b_{K}{∥}_{2}}{∥b_{1}{∥}_{2}}.$$

#### 3.1.1. Reconstructions with CST Data

“Case I” considers the numerical model in Figure 2 immersed inside “infinite” 90–10% glycerol-water mixture. A cylindrical blood target centered at $O_{tg}=($20 mm, 20 mm) with $\varepsilon_{r}=61.1-28.4j$, radius ρ=15 mm and height h=30 mm is inserted into the Zubal head phantom. Eight antennas are placed in an elliptical array configuration with semi-major and semi-minor axes equal to 100 mm and 85 mm, respectively. Two CST simulations are performed to obtain the scattered field data, with and without the target, to which we refer as WT and NT, respectively. The initial guess of FDTD model for reconstruction is chosen to be similar to the NT case CST model. The reconstruction area inside the brain is a cubical volume with height along the *Z*-axis between [10, 70] mm for the 3-D model.

**Figure 5:**
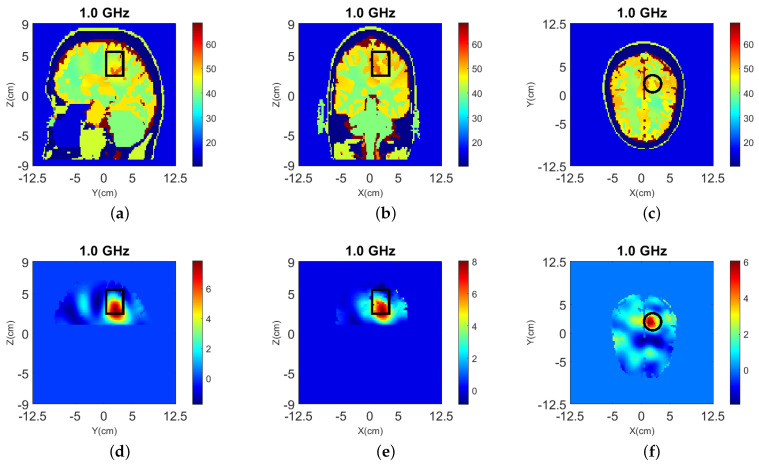
3-D reconstructions with simplified Green’s functions for “Case I” of Figure 2 at 1.0 GHz. (**a**–**c**) Relative permittivity $ℜ(\varepsilon_{r})$, and (**d**–**f**) contrast $ℜ(\delta_{\varepsilon })$ for the y-z, x-z, and x-y slices, respectively. A cylindrical eight-antenna array centered at the target height is used to produce the data.

The 3-D reconstruction results using the simplified Green’s function are shown in Figure 5, where the top shows the reconstructed relative permittivity $ℜ(\varepsilon_{r})$ and the bottom shows the contrast $ℜ(\delta_{\varepsilon })$ due to the target.

**Figure 6:**
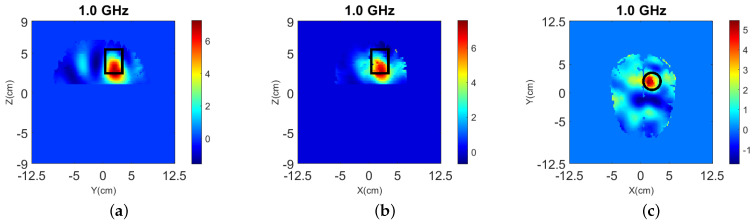
3-D reconstructions, same as Figure 5 but with vectorial Green’s function in (13), for “Case I” of Figure 2 at 1.0 GHz. (**a**–**c**) Relative permittivity $ℜ(\varepsilon_{r})$ for the y-z, x-z, and x-y slices, respectively.

We have also performed reconstructions using the vectorial Green’s function (13), and the reconstruction results of $ℜ(\delta_{\varepsilon })$ are shown in Figure 6 with errors RMSE=3.65, $e_{max}=20.42$, RE=0.27, which are close to the reconstructed values using the simplified Green’s function shown in the first row of Table 3.

This comparison suggests that the z-only approximation for the Green’s function does not affect the accuracy of the results. We thus argue that this formulation is sufficient to achieve similar accuracy with the more complete vector formulation of (13). To re-enforce this argument, we have performed another comparison of the two formulations for a more challenging imaging scenario in Section 3.2.

**Figure 7:**
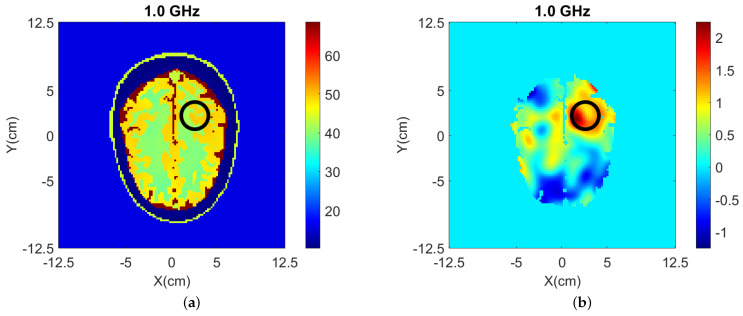
2-D reconstructions of: (**a**) relative permittivity $ℜ(\varepsilon_{r})$ and, (**b**) contrast $ℜ(\delta_{\varepsilon })$ for “Case I” of Figure 2 at 1.0 GHz.

The target is also detected in 2-D as shown in Figure 7, but the reconstructed values are lower.

Inaccuracies in these 2-D and 3-D results can be attributed to the complex brain structure which leads to a highly non-linear scattering problem, and to the mismatch between the CST model producing the data and the FDTD forward model of our DBIM-FISTA algorithm. For example, our FDTD solver models the antennas as point sources to avoid the additional computational complexity and much finer resolution required for modeling the full antenna structure, which leads to a model mismatch from the full antenna CST model. Moreover, the models in FDTD and CST have discrepancies in the Debye material properties due to their different computational environments. This includes errors in the head boundary between the CST (or experimental model) and the FDTD solver, due to discretization errors and the coarser resolution used in FDTD.

**Figure 8:**
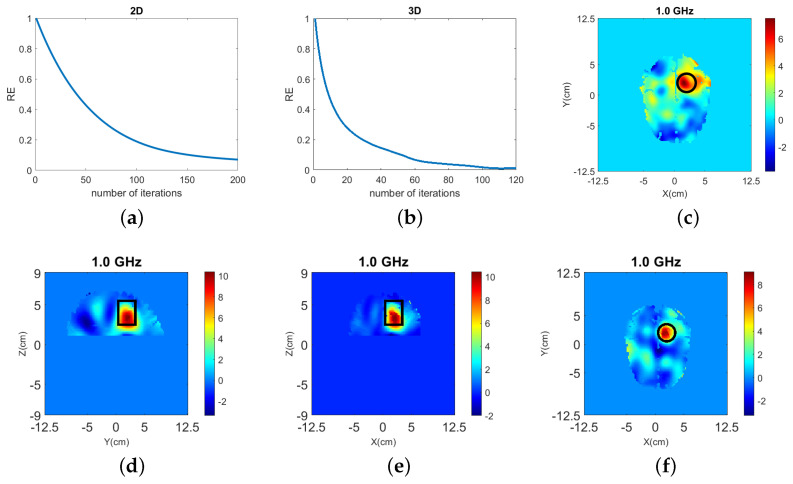
Reconstructions of “Case I” when RE converges: 2-D with 200 iterations and 3-D with 120 iterations. (**a**,**b**) Residual error plot for 2-D and 3-D, respectively. (**c**) Contrast $ℜ(\delta_{\varepsilon })$ for 2-D reconstruction. (**d**–**f**) Contrast $ℜ(\delta_{\varepsilon })$ for the y-z, x-z, and x-y slices of 3-D reconstruction, respectively.

To assess whether a fixed number of 20 iterations for both 2-D and 3-D algorithms leads to fair comparison, we have run Case I with a large number of iterations which can ensure that the residual error RE converges to almost a fixed value (see plots (a) and (b) in Figure 8). The numbers of iterations and errors for these cases are given in Table 3, and the resulting images are shown in Figure 8.

Relative to the reconstruction results with 20 DBIM iterations, reconstructions after a much greater number of iterations (120 in 3-D, 200 in 2-D) lead to rather modest reductions in the errors in Table 3, despite a significant decrease in the data residual RE. Importantly, the ratio between the 3-D and 2-D RMSE errors for the increased iterations is similar to the 20 iterations case, thereby allowing us to draw conclusions for 2-D vs. 3-D results using this limited fixed number. These observations and the results in Figure 8 suggest that using a fixed number of 20 iterations is sufficient for our comparison, although it does not represent complete convergence of the algorithms.

#### 3.1.2. Reconstructions with Experimental Data

**Figure 9:**
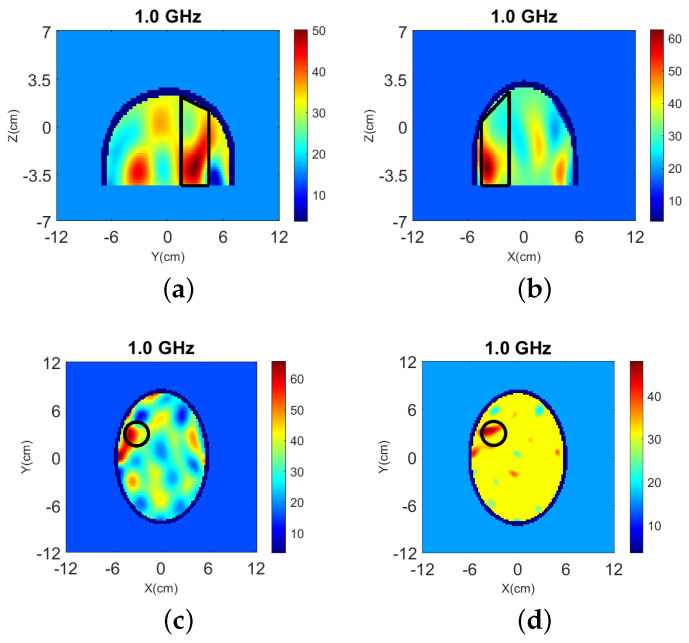
Reconstructions of $ℜ(\varepsilon_{r})$ for the experimental data at 1.0 GHz in 3-D: (**a**) y-z slice, (**b**) x-z slice, and (**c**) x-y slice, and in 2-D (**d**).

We conducted an experiment with the antenna array surrounding the lower half of the phantom shown in Figure 3. We inserted a cylindrical blood target with radius ρ=15 mm and permittivity $\varepsilon_{r}\approx 67.3-9.3j$ at 1.0 GHz into the phantom, centered at $O_{tg}=(-30$ mm, 30 mm) along the horizontal axes. Reconstructions in 3-D and for the 2-D slice defined by the antenna centers are shown in Figure 9, where the Debye parameters of the brain material $\varepsilon_{\infty }=20$, Δε=20 and $\sigma_{s}=0.147$ are used.

The 2-D reconstruction has less artifacts than the 3-D image along the same x-y slice, as it solves an inverse problem with a lot fewer unknowns. The y-z and x-z slices from the 3-D reconstructions show that the bottom part of the cylindrical target is detected more clearly than the upper half, as the eight-antenna ring is placed in the lower half. These images also suggest that reflections from the plastic container have created artifacts in the 3-D reconstruction images. Overall, both 2-D and 3-D algorithms have detected the target at the right position, albeit with artifacts that are more pronounced in 3-D for the axial slice. We have also performed reconstructions when the antenna ring is placed at different heights, and the results become worse and even fail to detect the target when the antenna ring is near the upper part of the head phantom due to that the curvature of the surface reflects signals into the air which are not captured by the antenna ring.

### 3.2. Imaging Performance with Limited Data

This subsection focuses on reconstructions of more challenging imaging scenarios. These include imaging with limited prior information, as well as detecting a target positioned in-between the antenna rings of a 3-D array. We compare 2-D and 3-D reconstructions for these cases to examine possible benefits of using the 3-D algorithm. Similar to the previous results, we have performed twenty DBIM iterations in all our reconstructions below.

#### 3.2.1. Reconstructions with Limited Prior Information

**Figure 10:**
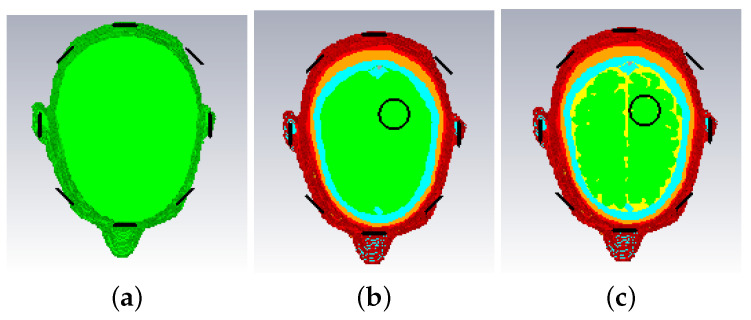
Cross-sectional view of models for “Case II.1” and “Case II.2”: (**a**) the NT model for both cases; (**b**) WT1 model for “Case II.1” where gray matter and CSF are replaced by white matter; (**c**) WT2 model for “Case II.2” where gray matter only is replaced by white matter.

**Figure 11:**
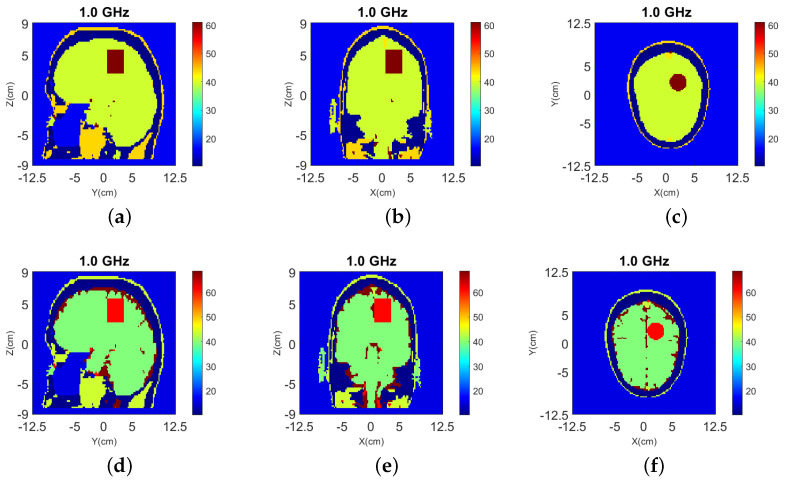
True values of $ℜ(\varepsilon_{r})$ of Case II.1 and II.2 at 1.0 GHz: (**a**) y-z slice, (**b**) x-z slice, and (**c**) x-y slice of Case II.1. (**d**) y-z slice, (**e**) x-z slice, and (**f**) x-y slice of Case II.2.

To investigate a scenario of limited prior information where only the boundary of the head is known, the model of Figure 2 was filled with white matter only for the NT case. Moreover, taking into account that the dielectric properties of gray and white matter are not very different (see Table 2), gray matter is replaced with white matter in Figure 2 to reduce the model complexity [41]. The resulting head model is then used in two WT cases (WT1 and WT2), “Case II.1” and “Case II.2”, which differ by the presence or absence of the CSF layer with $\varepsilon_{r}\approx 68.4-44.9j$. Cross-sectional views of the 3-D models for these cases are shown in Figure 10 while the true values of $ℜ(\varepsilon_{r})$ is shown in Figure 11.

**Figure 12:**
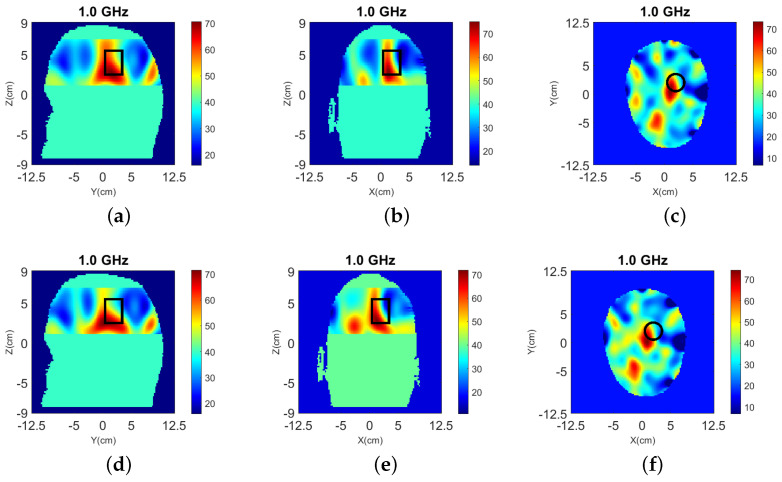
3-D reconstructions of $ℜ(\varepsilon_{r})$ at 1.0 GHz: (**a**) y-z slice, (**b**) x-z slice, and (**c**) x-y slice for Case II.1; (**d**) y-z slice, (**e**) x-z slice, and (**f**) x-y slice for Case II.2.

**Figure 13:**
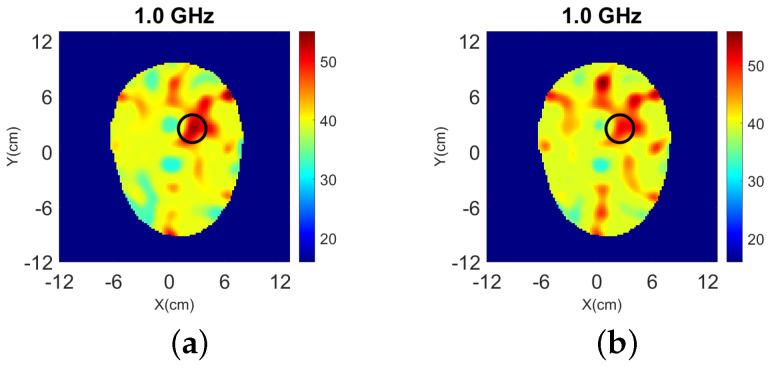
2-D reconstructions of $ℜ(\varepsilon_{r})$ at 1.0 GHz: (**a**) Case II.1, and (**b**) Case II.2. A cylindrical eight-antenna array centered at the target height is used to produce the data.

3-D and 2-D reconstruction results are shown in Figure 12 and Figure 13, respectively, with errors shown in Table 4.

The target is detected by both 2-D and 3-D algorithms, but there are significant image artifacts due to the skin, fat, and bone tissue regions which are not taken into account in the inverse model. These image artifacts are comparable in 2-D and 3-D, while the contrast in the estimated dielectric properties near the target is quite higher in the 3-D reconstructions. The RMSEs of both algorithms are similar but the 3-D has much larger $e_{max}$ values, which suggests that the 3-D algorithm may be more sensitive to limited prior information. Comparing the results of Cases II.1 and II.2, it can be concluded that not including a thin layer with high contrast such as CSF in the inverse model’s initial guess does not have a significant impact in the reconstructions.

#### 3.2.2. Reconstructions of Small Target at an Offset Height Using a Headband

**Figure 14:**
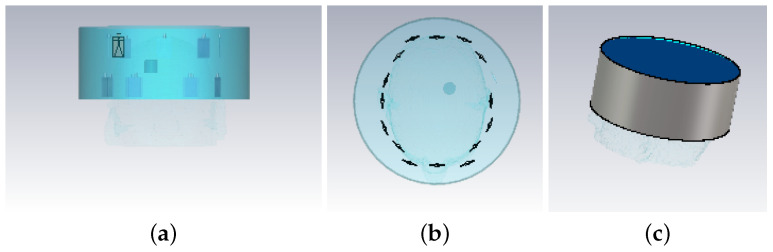
(**a**) Top and (**b**,**c**) side views of the setup used in Section 3.2. A headband is placed at the top of the head phantom, and sixteen antennas are placed in a two ring array inside the headband, with 8 antennas for each ring.

We consider another scenario in which a small target is placed in-between a two-ring array surrounding the model of Figure 2. The head phantom comprises white matter and includes a target at the same *x*-*y* position as previously, but with a smaller radius ρ=10 mm and height h=20 mm for the WT case. As the immersion liquid cannot extend infinitely in a practical scenario, we consider a headband of finite dimensions filled with the immersion liquid and surrounded by air. The setup is shown in Figure 14. The two rings are placed with an offset in the x-y plane, to obtain information from more angles and reduce coupling. The target covers an area between the x-y planes of the two rings only, as shown in Figure 14a.

We have considered different ways of applying our 2-D imaging algorithm to the data by the two-ring array by using: (1) the bottom ring data, (2) the top ring data, and (3) combined data from both rings as if they were on the same plane, effectively creating a sixteen-antenna array for the slice reconstructed in 2-D, which was selected as the slice of the target center.

**Figure 15:**
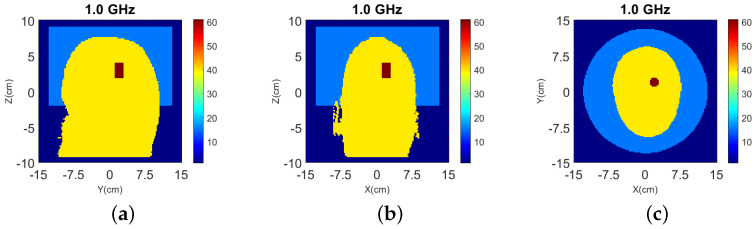
True values of $ℜ(\varepsilon_{r})$ of Case III at 1.0 GHz: (**a**) y-z slice, (**b**) x-z slice, and (**c**) x-y slice.

The head model for the cases considered in this subsection (Cases III.1, 2 and 3) is shown in Figure 15, while the headband used in each case is different.

We note that the “homogeneous white-matter” head model of Figure 15 is unrealistic, but it was selected to focus on comparing 3-D with 2-D results of more realistic arrays and smaller targets. To compare the errors of the target domain more clearly, we have also defined an RMSE in the selected target slice as RMSE–*T*.

“Case III.1” considers a headband of ρ=130 mm radius and h=110 mm height filled with the glycerol-water mixture. The antennas are very close to the air-liquid interface in all directions (top/bottom/side) for this case, resulting in strong reflections from the interface. The reflections can be taken into account by our 3-D FDTD model along all dimensions, while the 2-D model can only model the x-y boundary. In both models, of course, the wave reflections from the boundary will be different for the realistic CST model relative to its simplified version in FDTD, firstly because realistic antennas have been replaced by simple point sources.

**Figure 16:**
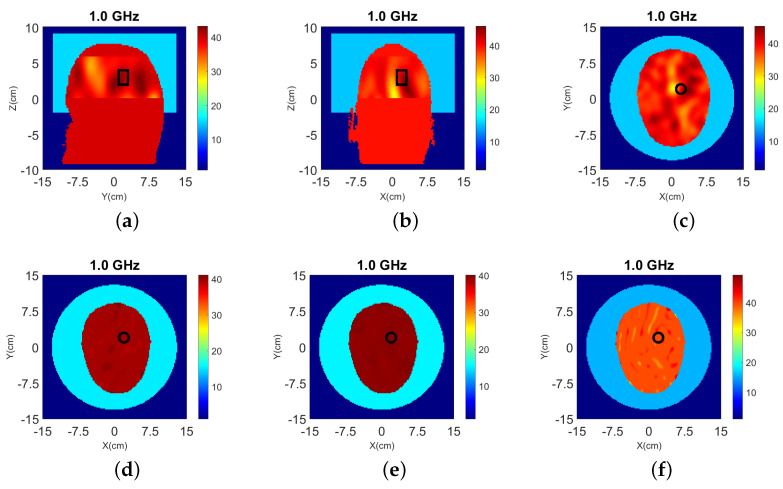
3-D (**a**–**c**) and 2-D (**d**–**f**) reconstructions of Case III.1 at 1.0 GHz: (**a**) y-z slice, (**b**) x-z slice, and (**c**) x-y slice of 3-D reconstructions. 2-D slice reconstructions (**d**) bottom ring, (**e**) top ring, and (**f**) both rings. The headband of Figure 14 is used to obtain the data.

Results from 3-D and 2-D reconstructions are shown in Figure 16.

Despite including the interface in the 3-D model, the reconstructed images suffer from strong artifacts near the interface, which suggest that the mismatch between the CST and FDTD model is significant. The target is reconstructed to some extent, but it is difficult to detect it with certainty. The 2-D reconstructions fail to detect the target completely regardless of whether we use data from the top, bottom, or both rings. This is not surprising given that the target is not aligned with any of these rings, and that the interface between air and the glycerol-water immersion liquid cannot be fully modeled in 2-D.

To examine the impact of this interface and improve detection performance, “Case III.2” considers the same headband as Case III.1, which is now surrounded by an additional layer of absorbing material. The headband layers are as follows: glycerol-water mixture, plastic, absorbing material (ECCOSORB MCS), and a metallic shield. Finally, “Case III.3” uses a a larger size (ρ=140 mm and h=120 mm) headband with the same materials as Case III.1. As with the absorbers in Case III.2, increasing the distance between the antennas and the interface with air can reduce the resulting reflections.

**Figure 17:**
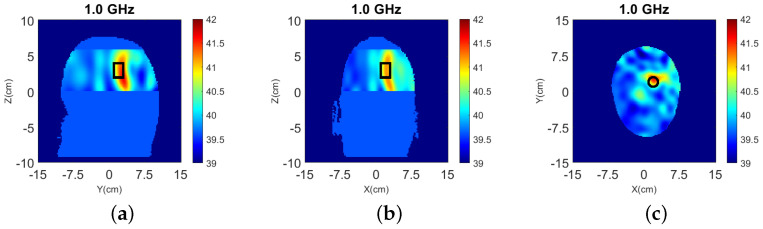
3-D reconstructions of $ℜ(\varepsilon_{r})$ for Case III.2 at 1.0 GHz: (**a**) y-z slice, (**b**) x-z slice, and (**c**) x-y slice. The headband of Figure 14 (with the addition of absorbing material) is used to obtain the data.

**Figure 18:**
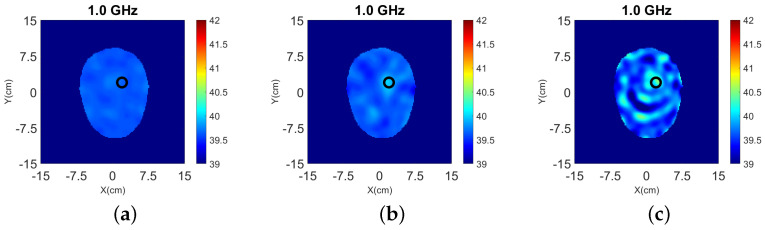
2-D Reconstructions of $ℜ(\varepsilon_{r})$ of Case III.2 for the x-y slice at 1.0 GHz using data from the: (**a**) bottom ring, (**b**) top ring, and (**c**) both rings. The headband of Figure 14 (with the addition of absorbing material) is used to obtain the data.

Results from 3-D and 2-D reconstructions at 1.0 GHz are shown in Figure 17 and Figure 18 for Case III.2. Note that we have limited the colorbar’s range of values in these images to increase the contrast. The 3-D reconstructions show that the target’s location and size is detected accurately with the use of the absorbers. The 2-D results, however, fail to distinguish the target from the noise.

**Figure 19:**
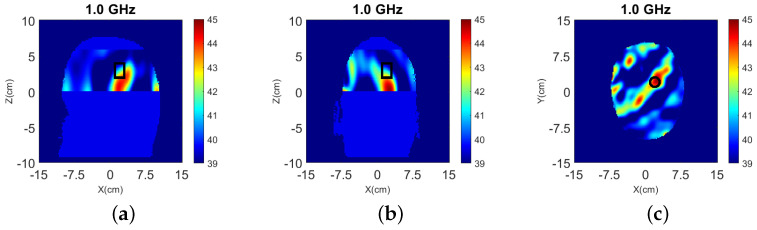
3-D reconstructions of $ℜ(\varepsilon_{r})$ for Case III.3 at 1.0 GHz: (**a**) y-z slice, (**b**) x-z slice, and (**c**) x-y slice. The headband of Figure 14 (with an increased distance between the array and the interface with air) is used to obtain the data.

**Figure 20:**
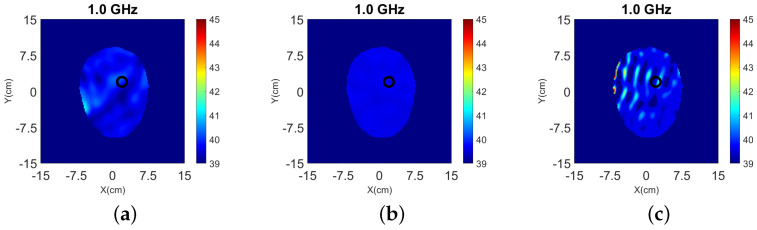
2-D Reconstructions of $ℜ(\varepsilon_{r})$ of Case III.3 for the x-y slice at 1.0 GHz using data from the: (**a**) bottom ring, (**b**) top ring, and (**c**) both rings. The headband of Figure 14 (with an increased distance between the array and the interface with air) is used to obtain the data.

Similarly, 3-D and 2-D reconstruction results for Case III.3 are presented in Figure 19 and Figure 20. Detection is improved from Case III.1, as the distance from the interface has been increased, but is less accurate than Case III.2 where the absorbing material was added. Similar to the other two cases, the 2-D imaging results in Figure 20 suggest that the algorithm fails to detect the target in all cases.

The RMSE, RMSE–*T*, $e_{max}$ and RE of Case III.1, 2 and 3 are shown in Table 5, where the 3-D reconstructions have a lower RMSE–*T* for all the three cases.

**Figure 21:**
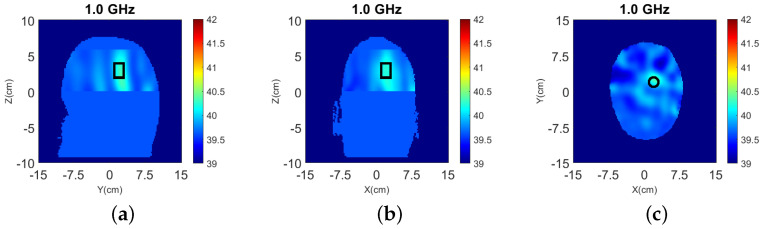
3-D reconstructions of $ℜ(\varepsilon_{r})$ for Case III.2 at 1.0 GHz: (**a**) y-z slice, (**b**) x-z slice, and (**c**) x-y slice, same as Figure 17 but with vectorial Green’s functions in (13).

Reconstructions of other cases with the vectorial Green’s function (13) have also been performed and Case III.2 is shown in Figure 21. The relative errors are RMSE=2.37, RMSE−T=20.89, $e_{max}=21.08$, RE=0.62, which again shows similar reconstruction quality with the simplified Green’s function even when the antenna array is placed at an offset height. This comparison confirms that the simplified Green’s function does not affect the 3-D reconstruction quality significantly, leading to equally accurate results for the scenarios considered in this paper.

Finally, we note that we have also performed reconstructions for additional scenarios related to Cases III.1–3, i.e., headbands with radii ρ=135 and 140 mm, with and without the absorbing layers. As expected, when the interface distance increases, detection accuracy and reconstruction quality improves. Moreover, the absorbing materials improve reconstructions further. Importantly, the 3-D imaging outperforms its 2-D counterpart in all these cases.

## 4. Discussion

We have developed and validated a computationally efficient 3-D DBIM algorithm for microwave head imaging. Our DBIM implementation uses the FISTA solver for the linear inverse problem, which has shown advantages over traditional CGLS solvers in our previous work [58]. The 3-D DBIM-FISTA algorithm relies on an in-house 3-D FDTD forward solver implemented on GPU, which is equally accurate but runs considerably faster than previous implementations with commercial software Acceleware. Our implementation combined this 3-D GPU-based FDTD solver with the inversion code on MATLAB via MEX functions.

Inspired by microwave head imaging applications, we used CST-calculated as well as experimental data to validate the 3-D DBIM-FISTA algorithm. We implemented a simplified 3-D inverse algorithm that considers a scalar Green’s function for linearly polarized antennas, such as the printed monopoles used by our system. Importantly, we showed that this approximation does not lead to worse performance than a vectorial Green’s function implementation for the considered head imaging scenarios. Our numerical studies employed different numerical head models in CST based on the Zubal head phantom. Our experimental study examined a simpler, homogeneous head model, with the purpose to validate the 3-D implementation rather than examining more complex head models.

Our results showed that reconstructions are of similar quality to those produced by a 2-D version of the algorithm for cases where the data processed by the 2-D imaging algorithm is of sufficient quality. To show that this may not always be possible, we considered cases where the target was not centered at the same transverse plane as the antenna ring and reflections from the interface of a headband with air were significant. Comparison of 3-D and 2-D reconstructions for these imaging scenarios showcased the advantages of imaging in 3-D. Moreover, these cases also demonstrated that terminating the imaging headband with absorbing material can improve drastically the 3-D array’s imaging performance.

Future work will focus further on reconstructions with experimental data inspired by the outcomes of this study. In particular, using prior information to improve reconstruction accuracy in realistic clinical settings requires further investigation. This prior information is not only required for dealing with the complexity of the brain’s heterogeneous tissues, but also to estimate accurately the head boundary, which constitutes the reconstruction domain of our MWT approach. Moreover, the issue of patient movement should also be investigated in clinical settings. Motion artefacts are an important issue in imaging techniques which require long data acquisition times such as MRI, and a MWT system must be designed to minimise their impact by ensuring very short data acquisition times.

## Figures and Tables

**Table 1:** Average running time for each antenna.

Resolution	Grid Size	Time
2 mm	150×150×100	31 s
1.5 mm	225×225×150	59 s
1 mm	300×300×200	176 s

**Table 2:** Debye parameters for the Zubal head phantom.

Tissue Type and Respective Color in Figure 2d	Permittivity at Infinite Frequency $\varepsilon_{\infty }$	Debye Dispersion Δε	Conductivity $\sigma_{s}$(S/m)
skin (red)	37.65	11.36	0.62
fat (orange)	8.61	2.92	0.08
bone (blue)	8.48	4.38	0.08
white matter (green)	35.89	6.73	0.45
gray matter (brown)	40.03	14.47	0.72
blood (black)	44.67	18.02	1.32
CSF (yellow)	66.08	4.61	2.34
dura (pink)	39.89	6.00	0.85

**Table 3:** Reconstruction errors for the cases considered in Section 3.1.1.

Simulation Name	Iteration	RMSE	$e_{\max}$	RE
Case I, 3-D	20	3.39	19.38	0.28
Case I, 2-D	20	4.11	20.46	0.72
Case I, 3-D	120	3.21	19.42	0.01
Case I, 2-D	200	3.71	19.03	0.07
Exp, 3-D	20	9.73	48.87	0.17
Exp, 2-D	20	7.31	36.23	0.73

**Table 4:** Reconstruction errors for the cases considered in Section 3.2.1.

Case No.	RMSE	$e_{\max}$	RE
Case II.1, 3-D	16.62	46.71	0.19
Case II.2, 3-D	18.75	62.01	0.51
Case II.1, 2-D	15.51	22.27	0.16
Case II.2, 2-D	18.01	21.68	0.50

**Table 5:** Reconstruction Errors for the cases considered in Section 3.2.2.

Case No.	RMSE	RMSE **–*T***	$e_{\max}$	RE
III.1, 3-D	3.05	20.14	22.79	0.57
III.1, 2-D bot	2.44	21.35	21.49	0.71
III.1, 2-D top	2.43	21.27	21.40	0.69
III.1, 2-D both	2.59	21.40	21.48	0.75
III.2, 3-D	2.30	20.02	20.70	0.70
III.2, 2-D bot	2.44	21.49	21.44	0.70
III.2, 2-D top	2.41	21.23	21.15	0.70
III.2, 2-D both	2.40	21.25	20.92	0.72
III.3, 3-D	2.57	20.08	21.14	0.47
III.3, 2-D bot	2.43	21.17	21.59	0.70
III.3, 2-D top	2.43	21.36	21.40	0.70
III.3, 2-D both	2.59	21.40	21.48	1.49

## Data Availability

Not applicable.

## Abbreviations

The following abbreviations are used in this manuscript:

DBIM	distorted Born iterative method
GPU	graphic processing unit
3-D	three-dimensional
2-D	two-dimensional
MWT	microwave tomography
MWI	microwave imaging
FDTD	finite difference time domain
EM	electromagnetic
DDA	discrete dipole approximation
GN	Gauss-Newton
FEM	finite element method
CSI	contrast source inversion
VIE	volume integral equation
TSVD	truncated singular value decomposition
FISTA	fast iterative shrinkage/thresholding algorithm
CG	conjugate gradient
FFT	fast Fourier transform
TR	transmitter–receiver
CPML	convolutional perfectly matched layer
*RMSE*	root mean square error
*RE*	residual error

## References

[1] Semenov S. (2009). Microwave tomography: Review of the progress towards clinical applications. Philos. Trans. R. Soc. A.

[2] Grzegorczyk T.M., Meaney P.M., Kaufman P.A., Paulsen K.D. (2012). Fast 3-D tomographic microwave imaging for breast cancer detection. IEEE Trans. Med. Imag..

[3] Winters D.W., Shea J.D., Kosmas P., Van Veen B.D., Hagness S.C. (2009). Three-Dimensional Microwave Breast Imaging: Dispersive Dielectric Properties Estimation Using Patient-Specific Basis Functions. IEEE Trans. Med. Imag..

[4] Hopfer M., Planas R., Hamidipour A., Henriksson T., Semenov S. (2017). Electromagnetic Tomography for Detection, Differentiation, and Monitoring of Brain Stroke: A Virtual Data and Human Head Phantom Study. IEEE Antennas Propag. Mag..

[5] Kosmas P., Crocco L. (2019). Introduction to Special Issue on “Electromagnetic Technologies for Medical Diagnostics: Fundamental Issues, Clinical Applications and Perspectives. Diagnostics.

[6] Gabriel S., Lau R., Gabriel C. (1996). The dielectric properties of biological tissues: II. Measurements in the frequency range 10 Hz to 20 GHz. Phys. Med. Biol..

[7] Schwan H., Foster K. (1989). Dielectric properties of tissues and biological materials: A critical review. Crit. Rev. Biomed. Eng..

[8] Lazebnik M., McCartney L., Popovic D., Watkins C.B., Lindstrom M.J., Harter J., Sewall S., Magliocco A., Booske J.H., Okoniewski M. (2007). A large-scale study of the ultrawideband microwave dielectric properties of normal breast tissue obtained from reduction surgeries. Phys. Med. Biol..

[9] Sugitani T., Kubota S.i., Kuroki S.i., Sogo K., Arihiro K., Okada M., Kadoya T., Hide M., Oda M., Kikkawa T. (2014). Complex permittivities of breast tumor tissues obtained from cancer surgeries. Appl. Phys. Lett..

[10] Cheng Y., Fu M. (2018). Dielectric properties for non-invasive detection of normal, benign, and malignant breast tissues using microwave theories. Thorac. Cancer.

[11] Kaufman Z., Paran H., Haas I., Malinger P., Zehavi T., Karni T., Pappo I., Sandbank J., Diment J., Allweis T. (2016). Mapping breast tissue types by miniature radio-frequency near-field spectroscopy sensor in ex-vivo freshly excised specimens. BMC Med. Imaging.

[12] Martellosio A., Pasian M., Bozzi M., Perregrini L., Mazzanti A., Svelto F., Summers P.E., Renne G., Preda L., Bellomi M. (2016). Dielectric properties characterization from 0.5 to 50 GHz of breast cancer tissues. IEEE Trans. Microw. Theory Tech..

[13] Salahuddin S., Porter E., Meaney P.M., O’Halloran M. (2017). Effect of logarithmic and linear frequency scales on parametric modelling of tissue dielectric data. Biomed. Phys. Eng. Express.

[14] Scapaticci R., Di Donato L., Catapano I., Crocco L. (2012). A feasibility study on microwave imaging for brain stroke monitoring. Prog. Electromagn. Res..

[15] Isernia T., Pascazio V., Pierri R. (1997). A nonlinear estimation method in tomographic imaging. IEEE Trans. Geosci. Remote Sens..

[16] Isernia T., Pascazio V., Pierri R. (2001). On the local minima in a tomographic imaging technique. IEEE Trans. Geosci. Remote Sens..

[17] Semenov S.Y., Corfield D.R. (2008). Microwave tomography for brain imaging: Feasibility assessment for stroke detection. Int. J. Antennas Propag..

[18] Gilmore C., Mojabi P., Zakaria A., Ostadrahimi M., Kaye C., Noghanian S., Shafai L., Pistorius S., LoVetri J. (2009). A wideband microwave tomography system with a novel frequency selection procedure. IEEE Trans. Biomed. Eng..

[19] Yu C., Yuan M., Stang J., Bresslour E., George R.T., Ybarra G.A., Joines W.T., Liu Q.H. (2008). Active microwave imaging II: 3-D system prototype and image reconstruction from experimental data. IEEE Trans. Microw. Theory Tech..

[20] Meaney P.M., Fanning M.W., Li D., Poplack S.P., Paulsen K.D. (2000). A clinical prototype for active microwave imaging of the breast. IEEE Trans. Microw. Theory Tech..

[21] Gilmore C., Zakaria A., LoVetri J., Pistorius S. (2013). A study of matching fluid loss in a biomedical microwave tomography system. Med. Phys..

[22] Semenov S.Y., Svenson R.H., Bulyshev A.E., Souvorov A.E., Nazarov A.G., Sizov Y.E., Posukh V.G., Pavlovsky A., Repin P.N., Starostin A.N. (2002). Three-dimensional microwave tomography: Initial experimental imaging of animals. IEEE Trans. Biomed. Eng..

[23] Rubæk T., Mohr J.J. (2016). Microwave tomography. An Introduction to Microwave Imaging for Breast Cancer Detection.

[24] Fang Q., Meaney P.M., Paulsen K.D. (2009). Viable three-dimensional medical microwave tomography: Theory and numerical experiments. IEEE Trans. Antennas Propag..

[25] Shea J.D., Kosmas P., Hagness S.C., Van Veen B.D. (2010). Three-dimensional microwave imaging of realistic numerical breast phantoms via a multiple-frequency inverse scattering technique. Med. Phys..

[26] Colgan T.J., Hagness S.C., Van Veen B.D. (2015). A 3-D level set method for microwave breast imaging. IEEE Trans. Biomed. Eng..

[27] Kurrant D., Fear E., Baran A., LoVetri J. (2017). Integrating prior information into microwave tomography part 2: Impact of errors in prior information on microwave tomography image quality. Med. Phys..

[28] Zwamborn P., Van Den Berg P.M. (1992). The three dimensional weak form of the conjugate gradient FFT method for solving scattering problems. IEEE Trans. Microw. Theory Tech..

[29] Giannakopoulos I.I., Litsarev M.S., Polimeridis A.G. (2019). Memory Footprint Reduction for the FFT-Based Volume Integral Equation Method via Tensor Decompositions. IEEE Trans. Antennas Propag..

[30] Asefi M., Baran A., LoVetri J. (2019). An Experimental Phantom Study for Air-Based Quasi-Resonant Microwave Breast Imaging. IEEE Trans. Microw. Theory Tech..

[31] Fhager A., Padhi S.K., Howard J. (2009). 3D Image Reconstruction in Microwave Tomography Using an Efficient FDTD Model. IEEE Antennas Wirel. Propag. Lett..

[32] Ireland D., Abbosh A. (2013). Modeling Human Head at Microwave Frequencies Using Optimized Debye Models and FDTD Method. IEEE Trans. Antennas Propag..

[33] Munawar Qureshi A., Mustansar Z., Maqsood A. (2016). Analysis of microwave scattering from a realistic human head model for brain stroke detection using electromagnetic impedance tomography. Prog. Electromagn. Res..

[34] Fedeli A., Schenone V., Randazzo A., Pastorino M., Henriksson T., Semenov S. (2020). Nonlinear S-parameters inversion for stroke imaging. IEEE Trans. Microw. Theory Tech..

[35] Zamani A., Abbosh A.M., Mobashsher A.T. (2016). Fast Frequency-Based Multistatic Microwave Imaging Algorithm With Application to Brain Injury Detection. IEEE Trans. Microw. Theory Tech..

[36] Alqadami A.S.M., Zamani A., Trakic A., Abbosh A. (2021). Flexible Electromagnetic Cap for Three-Dimensional Electromagnetic Head Imaging. IEEE. Trans. Biomed. Eng..

[37] Trakic A., Brankovic A., Zamani A., Nguyen-Trong N., Mohammed B., Stancombe A., Guo L., Bialkowski K., Abbosh A. (2020). Expedited Stroke Imaging With Electromagnetic Polar Sensitivity Encoding. IEEE Trans. Antennas Propag..

[38] Tournier P.H., Bonazzoli M., Dolean V., Rapetti F., Hecht F., Nataf F., Aliferis I., El Kanfoud I., Migliaccio C., de Buhan M. (2017). Numerical Modeling and High-Speed Parallel Computing: New Perspectives on Tomographic Microwave Imaging for Brain Stroke Detection and Monitoring. IEEE Antennas Propag. Mag..

[39] Tobon Vasquez J.A., Scapaticci R., Turvani G., Bellizzi G., Rodriguez-Duarte D.O., Joachimowicz N., Duchêne B., Tedeschi E., Casu M.R., Crocco L. (2020). A prototype microwave system for 3D brain stroke imaging. Sensors.

[40] Lu P., Córcoles J., Kosmas P. Non-linear Microwave Imaging Using Fast Iterative Shrinkage Thresholding. Proceedings of the 2019 PhotonIcs & Electromagnetics Research Symposium-Spring (PIERS-Spring).

[41] Lu P., Ahsan S., Kosmas P. Preliminary Study on the Feasibility of Reconstructing Anatomically Complex Numerical Brain Phantoms with Limited Prior Information. Proceedings of the 2020 XXXIIIrd General Assembly and Scientific Symposium of the International Union of Radio Science.

[42] Acceleware Ltd. Acceleware FDTD Solver. https://www.acceleware.com/fdtd-solvers.

[43] Beck A., Teboulle M. (2009). A Fast Iterative Shrinkage-Thresholding Algorithm for Linear Inverse Problems. SIAM J. Imaging Sci..

[44] Ambrosanio M., Pascazio V. Compressive Sensing for Breast Microwave Imaging. Proceedings of the 2018 40th Annual International Conference of the IEEE Engineering in Medicine and Biology Society (EMBC).

[45] Zubal I.G., Harrell C.R., Smith E.O., Rattner Z., Gindi G., Hoffer P.B. (1994). Computerized three-dimensional segmented human anatomy. Med. Phys..

[46] Chew W.C. (1995). Preliminary background. Waves and Fields in Inhomogenous Media.

[47] Shea J.D., Kosmas P., Veen B.D.V., Hagness S.C. (2010). Contrast-enhanced microwave imaging of breast tumors: A computational study using 3D realistic numerical phantoms. Inverse Probl..

[48] Bulyshev A.E., Souvorov A.E., Semenov S.Y., Svenson R.H., Nazarov A.G., Sizov Y.E., Tatsis G.P. (2000). Three-dimensional microwave tomography. Theory and computer experiments in scalar approximation. Inverse Probl..

[49] Chi J., Liu F., Weber E., Li Y., Crozier S. (2011). GPU-accelerated FDTD modeling of radio-frequency field–tissue interactions in high-field MRI. IEEE. Trans. Biomed. Eng..

[50] NVIDIA Corporation CUDA Programming Guide. https://docs.nvidia.com/cuda/cuda-c-programming-guide/index.html.

[51] Miao Z., Kosmas P. (2017). Multiple-frequency DBIM-TwIST algorithm for microwave breast imaging. IEEE Trans. Antennas Propag..

[52] Hasgall P.A., Neufeld E., Gosselin M.C., Klingenböck A., Kuster N., Hasgall P., Gosselin M. IT’IS Database for Thermal and Electromagnetic Parameters of Biological Tissues. https://itis.swiss/virtual-population/tissue-properties/.

[53] Ahsan S., Guo Z., Miao Z., Sotiriou I., Koutsoupidou M., Kallos E., Palikaras G., Kosmas P. (2018). Design and Experimental Validation of a Multiple-Frequency Microwave Tomography System Employing the DBIM-TwIST Algorithm. Sensors.

[54] Karadima O., Rahman M., Sotiriou I., Ghavami N., Lu P., Ahsan S., Kosmas P. (2020). Experimental Validation of Microwave Tomography with the DBIM-TwIST Algorithm for Brain Stroke Detection and Classification. Sensors.

[55] Guo W., Ahsan S., He M., Koutsoupidou M., Kosmas P. Printed Monopole Antenna Designs for a Microwave Head Scanner. Proceedings of the 2018 18th Mediterranean Microwave Symposium (MMS).

[56] Bucci O., Isernia T. (1997). Electromagnetic inverse scattering: Retrievable information and measurement strategies. Radio Sci..

[57] Kim E., Mohammadi C.T., Asefi M., Lovetri J., Jeffrey I., Gilmore C. (2022). Imaging and Calibration of Electromagnetic Inversion Data With a Single Data Set. IEEE Open J. Antennas Propag..

[58] Lu P., Córcoles J., Kosmas P. (2020). Enhanced FEM-based DBIM approach for two-dimensional microwave imaging. IEEE Trans. Antennas Propag..

